# Histological Evaluation of Porous Additive-Manufacturing Titanium Artificial Bone in Rat Calvarial Bone Defects

**DOI:** 10.3390/ma14185360

**Published:** 2021-09-17

**Authors:** Naoko Imagawa, Kazuya Inoue, Keisuke Matsumoto, Michi Omori, Kayoko Yamamoto, Yoichiro Nakajima, Nahoko Kato-Kogoe, Hiroyuki Nakano, Phuc Thi Minh Le, Seiji Yamaguchi, Takaaki Ueno

**Affiliations:** 1 Department of Dentistry and Oral Surgery, Division of Medicine for Function and Morphology of Sensor Organs, Faculty of Medicine, Osaka Medical and Pharmaceutical University, 2-7 Daigakumachi, Takatsuki, Osaka 569-8686, Japan; naoko.imagawa@ompu.ac.jp (N.I.); keisuke.matsumoto@ompu.ac.jp (K.M.); michi.omori@ompu.ac.jp (M.O.); kayoko.yamamoto@ompu.ac.jp (K.Y.); n4160@ompu.ac.jp (Y.N.); nahoko.kogoe@ompu.ac.jp (N.K.-K.); hiroyuki.nakano@ompu.ac.jp (H.N.); ueno@ompu.ac.jp (T.U.); 2 Department of Biomedical Science, College of Life and Health Sciences, Chubu University, 1200 Matsumoto-cho, Kasugai, Aichi 487-8501, Japan; minhphuc@isc.chubu.ac.jp (P.T.M.L.); sy-esi@isc.chubu.ac.jp (S.Y.)

**Keywords:** additive-manufacturing titanium artificial bone, histological observation, mixed-acid

## Abstract

Jaw reconstruction using an additive-manufacturing titanium artificial bone (AMTAB) has recently attracted considerable attention. The synthesis of a titanium artificial bone is based on three-dimensional computed tomography images acquired before surgery. A histological evaluation of porous AMTAB (pAMTAB) embedded in rat calvarial bone defects was conducted. This study examined three groups: rats implanted with mixed-acid and heat-treated pAMTAB, rats implanted with untreated pAMTAB, and rats with no implant. In both pAMTAB groups, bone defects were created in rat calvarial bones using a 5-mm trephine bar, followed by pAMTAB implantation. The pAMTAB was fixed to the defect using the fitting force of the surrounding bones. The rats were sacrificed at 4, 8, and 16 weeks after implantation, and the skull was dissected. Undecalcified ground slides were prepared and stained with Villanueva Goldner. Compared with the no implant control group, both pAMTAB groups exhibited new bone formation inside the defect, with greater bone formation in the mixed-acid and heat-treated pAMTAB group than in the untreated pAMTAB group, but the difference was not significant. These data suggest that pAMTAB induces bone formation after implantation in bone defects. Bone formation appears to be enhanced by prior mixed-acid and heat-treated pAMTAB.

## 1. Introduction

Recent innovative developments in computer-assisted technologies involving computed tomography (CT) data have revolutionized the fields of jaw bone reconstruction and dental implants [1,2,3]. We previously reported the usefulness of bone augmentation in dental implants using additive-manufacturing titanium (Ti) artificial membranes [4]; the artificial membranes were able to fit the bone defect easier and shorten operation times.

Jaw reconstruction surgery is required when defects in the jaw bone result from a tumor or trauma. Such surgery involves the adaptation of a reconstruction plate to the jaw bone. However, most fixation plates used for surgery have a tabular shape, and the surgeon must physically manipulate the plate to conform to the shape of the jaw bone. This raises several concerns, such as extending the duration of the surgery and weakening of the plate’s mechanical strength due to repetitive bending. In addition, the success of the surgery varies depending on the surgeon’s skill level.

A porous additive-manufacturing Ti artificial bone (pAMTAB) prepared via three-dimensional simulation could be used to overcome the abovementioned concerns. Our method of preparing a suitable artificial bone is based on the patient’s preoperative CT data and allows for the preoperative adaptation of the plate, thus shortening the duration of surgery. In addition, the passive fit between the plate and the jaw bone prevents the weakening of the plate’s mechanical strength due to repetitive bending. Ideally, artificial bone used in jaw bone reconstruction should have a porous structure and be biocompatible with the patient’s bone. Matsushita et al. [5] previously reported that pAMTAB, prepared by simulating the cancellous bone structure, exhibited the same biocompatibility as the cancellous bone [6]. Furthermore, Kokubo et al. [7] reported that Ti exhibits strong bone bonding after the surface is subjected to mixed acid and heat treatment. Yamamoto et al. reported that bone formation ability in various Ti surface modifications and mixed acid and heat treatment resulted in high bone formation ability. Some studies have reported that mixed acid and heat treatment increased the bone bonding of Ti. In addition, we recently reported that mixed-acid and heat-treated AMTAB exhibited stronger bone bonding than untreated AMTAB in the rat calvarial bone [8]. Bone-bonding strength was evaluated between the surface of rat calvarial bone and pAMTAB. However, bone formation in the pAMTAB was not evaluated. There are no studies evaluating bone formation following implantation of mixed-acid and heat-treated pAMTAB in bone defects.

Therefore, this study aimed to histologically examine whether pAMTAB is a suitable substitute for a cancellous bone-like structure. In addition, we evaluated whether mixed-acid and heat-treated pAMTAB enhances its ability to promote bone formation.

## 2. Materials and Methods

### 2.1. Implant Preparation

**Figure 1:**
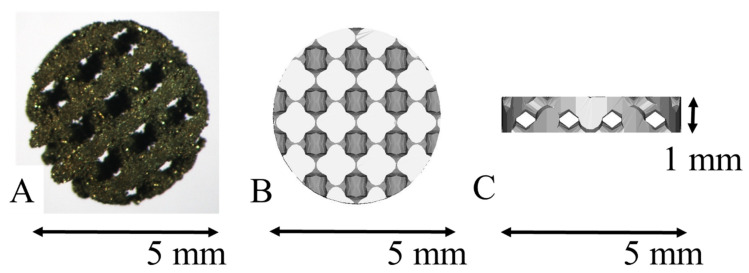
Porous additive-manufacturing titanium artificial bone (pAMTAB) images. (**A**) The actual pAMTAB has a width of 5.0 mm and a height of 1.0 mm. (**B**) Upper view of the pAMTAB structure. (**C**) Lateral view of the pAMTAB structure.

pAMTABs were prepared using a layered manufacturing process, with a diameter of 5 mm, height of 1 mm, pore diameter of 0.4 mm, support diameter of 0.5 mm, and porosity of 46%. The simulated pAMTABs were converted into data in a Standard Triangulation Language (STL) format. The pAMTABs were prepared using a selective laser melting (SLM) device (EOSM290, NTT DATA, Tokyo, Japan) as an additive-manufacturing machine. The pAMTABs were placed into a metal laminate molding apparatus, and the molding position was determined within the modeling range using onboard internal software. Pure Ti powder (diameter = 45 μm) was scattered onto the shaping sheet and moved with a laser light, following the two-dimensional data for each layer with respect to the molding position and modeling shape. Only the powder irradiated by the laser light was selectively melted and coagulated into a 30-µm-thick layer. The specimens for testing were prepared by repeating the procedure described above (Figure 1).

### 2.2. Chemical Treatment (Mixed Acid and Heat Treatment) of the Titanium Surface

Ultrasonically cleaned Ti mesh samples were immersed in a mixed-acid solution (1:1 mass mixture of 66.3% sulfuric acid and 10.6% hydrochloric acid), followed by immersion for 1 h in an oil bath maintained at 70 °C. During mixed-acid immersion, the samples were agitated at a rate of 120 times/min to promote uniform treatment of the surface. The samples were then gently rinsed by immersion in ultrapure water for 1 h, then heated in air from room temperature to 600 °C at a rate of 5 °C/min, and maintained at 600 °C for 1 h. After heating, the samples were allowed to cool naturally in a furnace to complete the mixed acid and heat treatment.

### 2.3. Evaluation of Surface Roughness

Surface roughness parameters were assessed using a Mitutoyo Surftest SV-2000 roughness gauge (Mitutoyo America Corp., Aurora, IL, USA). Surface roughness was evaluated by measuring five random positions on each SLM-Ti and SLM-Ti mixed-acid and heat-treated surfaces over a total length of 8000 µm.

### 2.4. Water Contact Angle Determination

The wettability of the SLM-Ti samples was determined by measuring the water contact angle. A 4-µL droplet of ultrapure water was deposited on the surface using a micropipette, and the contact angle was measured within a few seconds after the droplet was applied. The shape of the water droplet was observed, and the droplet was then photographed. The contact angle was calculated from a photographic image.

### 2.5. Surgical Procedure

A total of 45 female Sprague–Dawley rats (7 weeks old) were used in this study. The rats were divided into three groups: mixed-acid and heat-treated pAMTAB group (4, 8, and 16 weeks after surgery, five rats each), untreated pAMTAB group (4, 8, and 16 weeks after surgery, five rats each), and no implant group (4, 8, and 16 weeks after surgery, five rats each).

**Figure 2:**
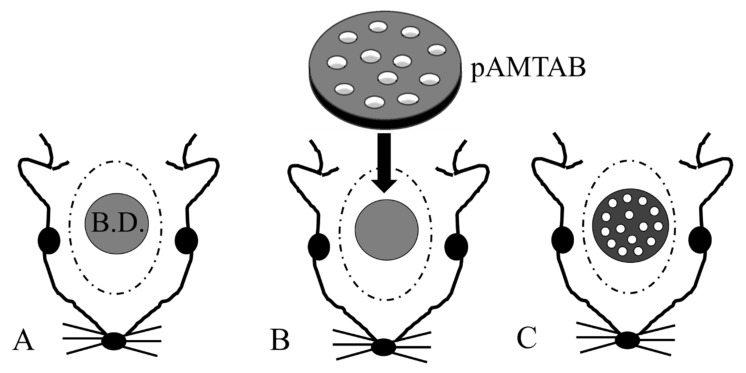
Photograph of implant placement. (**A**) A defect is created in a rat calvarial bone using a trephine bar (5 mm diameter). B.D.: bone defect. (**B**) The pAMTAB is inserted into the defect. (**C**) The pAMTAB is rigidly implanted in the bone defect.

Figure 2 shows a flowchart describing the animal experiments. The rats were sedated with isoflurane, and an incision was made in the scalp. The periosteum was then peeled away, and a bone defect in the calvarial bone reaching the dura mater was created using a trephine bar (φ5 mm in diameter). The appropriate pAMTAB (mixed acid and heat treated or untreated) was implanted in the bone defect, and proper fixation of the pAMTAB within the bone defect was confirmed based on the fitting force. The periosteum was sutured using 4-0 polyglactin (Vicryl^®^, Johnson & Johnson K. K., Tokyo, Japan), and the skin was sutured using 3-0 black silk thread (Mani^®^, Mani, Tochigi, Japan). In the control group, the bone defect was sutured immediately after creation. At 4, 8, and 16 weeks after surgery, the rats were euthanized with an overdose of isoflurane, the calvarial bone was harvested from each animal, and specimens for further examination were prepared. The protocol of this study was reviewed and approved by the Animal Research Committee of Osaka Medical College (approval number: 30014).

### 2.6. Radiography

Micro-CT imaging using a LaTheta LCT200^®^ instrument (HITACHI, Tokyo, Japan) was used to monitor for possible detachment of the pAMTAB after implantation and before euthanization to confirm that the pAMTAB did not deviate from the calvarial bone. Imaging was performed at a tube voltage of 50 kV, tube current of 2 mA, pixel size of 48 mm, and slice thickness of 96 mm.

### 2.7. Histological Analyses

Tissue samples were fixed in 20% formalin dehydrated/degreased using ethanol and acetone and then immersed in methylmethacrylate to prepare resin blocks. Frontal sections were prepared by cutting the block using a micro-cutting machine (BS-300CL/EXAKT^®^, Meiwafosis Co., Ltd., Tokyo, Japan) and a micro-grinding machine (MG-400CS/EXAKT^®^, Meiwafosis Co., Ltd.), such that the sections passed through the center of the bone defect. Ground slides (40 μm thick) were prepared, treated with xylene to remove resin and Villanueva Goldner stain, and then sealed. The frontal section of the bone defect area was observed under an optical microscope (Nikon ECLIPSE Ci^®^, Nikon, Tokyo, Japan).

### 2.8. Determination of the Amount of Bone Formed

**Figure 3:**
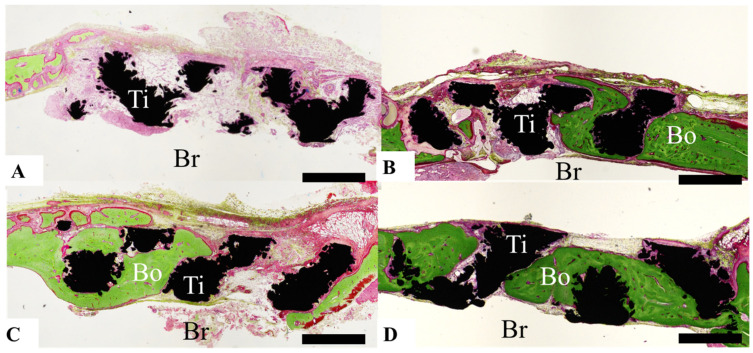
Photographs illustrating the scoring of the amount of bone formed based on histological analysis. (**A**) Score 0: Bone formation is virtually nonexistent. (**B**) Score 1: Bone has formed, but does not reach the center of the bone defect, or bone formation is observed only at the center of the bone defect. (**C**) Score 2: Bone formation extends past the center of the bone defect, but areas of bone defect remain. (**D**) Score 3: Almost the entire bone defect area is covered with bone. Scale bar indicates 1000 µm.

Healing of calvarial bone defects was assessed by determining the amount of bone formed from the bone defect margin, expressed as scores according to the findings shown in Figure 3, as follows: Score 0, virtually no bone formation was observed (Figure 3A); score 1, bone formation was observed, but it did not reach the center of the bone defect, or bone formation was observed only at the center of the bone defect (Figure 3B); score 2, although bone formation extended past the center of the defect, bone was still absent in some areas (Figure 3C); and score 3, almost the entire bone defect area was covered with bone (Figure 3D). The bone formation scores of each group were compared with the total amount. Two different researchers evaluated bone formation and confirmed the reproducibility of the score.

### 2.9. Bone Volume Ratio (Percent)

**Figure 4:**
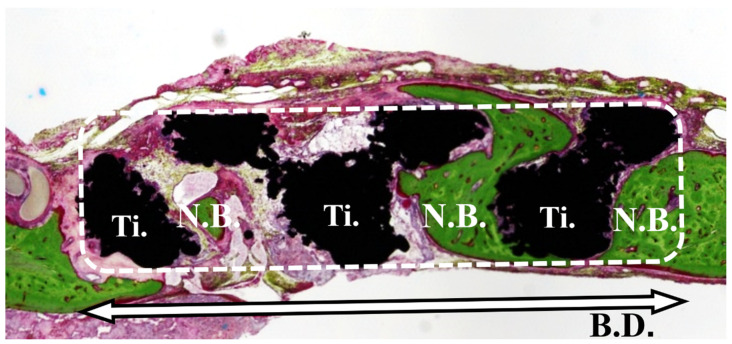
Bone volume ratio (%). Bone occupancy was calculated as the amount of newly formed bone in the space inside the bone defect.

The bone volume ratio (BV%) was defined as the amount of newly formed bone in the space located within the bone defect area (Figure 4) and determined by evaluation under an optical microscope (ECLIPSE Ci^®^, Nikon). BV% was calculated according to the following formula: BV% = total area of the newly formed bone (N.B.)/total area of the bone defect (B.D.) − total area of the Ti × 100.

### 2.10. Statistical Analyses

The data for each group were analyzed by one-way analysis of variance and Tukey’s multiple comparison test using JMP^®^ software (SAS Institute Inc., Cary, NC, USA). *p* values < 0.05 were considered significant.

## 3. Results

### 3.1. Evaluation of Surface Roughness

**Figure 5:**
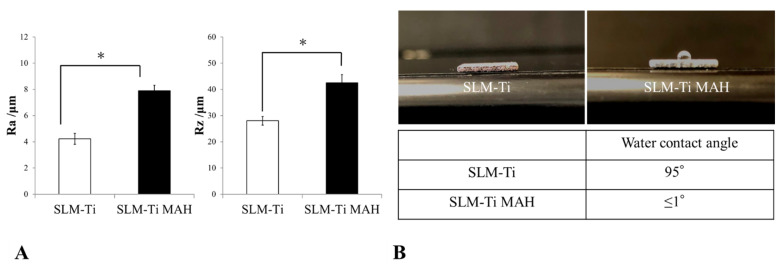
(**A**) Measurement of surface roughness using a Mitutoyo Surftest SV-2000 roughness gauge. Surface roughness parameters of selective laser melting-titanium before and after treatment with mixed acid and heat are shown. Ra: average peak to valley distance; Rz: highest peak to lowest valley distance. * *p* < 0.05. (**B**) Determination of the water contact angle. The shape of the water droplet was observed and photographed. The contact angle was calculated from the photographic image.

Figure 5 shows surface roughness parameters of SLM-Ti before and after treatment with mixed acid and heat. Average peak to valley distance (Ra) and highest peak to lowest valley distance (Rz) values were significantly higher in the SLM-Ti mixed-acid and heat-treatment group than in the untreated SLM-Ti group.

### 3.2. Water Contact Angle Determination

The shape of a water droplet deposited on the surface of each SLM-Ti was observed, and the contact angle was calculated from the photographs of each droplet, as shown in Figure 5. The water contact angle on untreated SLM-Ti was approximately 95°, whereas the angle on SLM-Ti subjected to mixed acid and heat treatment was ≤1°.

### 3.3. Radiography

**Figure 6:**
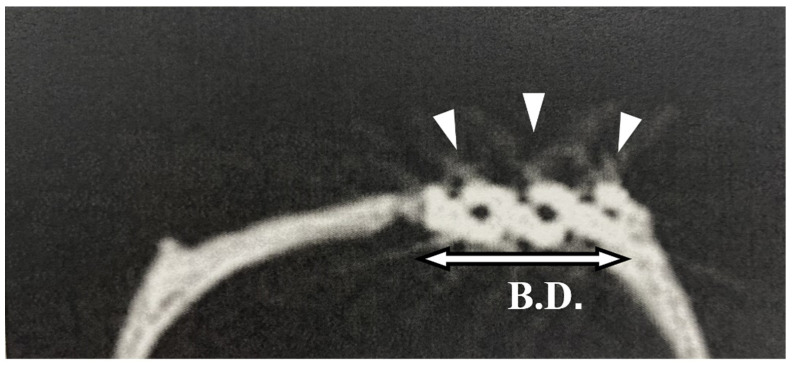
Micro-computed tomography image of a placed implant. The porous additive-manufacturing titanium artificial bone (pAMTAB) is observed in the calvarial bone defect; there is no deviation. pAMTAB is denoted by arrow heads.

The pAMTAB remained fixed inside the bone defect in all pAMTAB groups. None of the rats exhibited dislocation of the pAMTAB from the calvarial bones (Figure 6) Bone formation was not observed in the CT image due to the slight halation.

### 3.4. Histological Analyses

**Figure 7:**
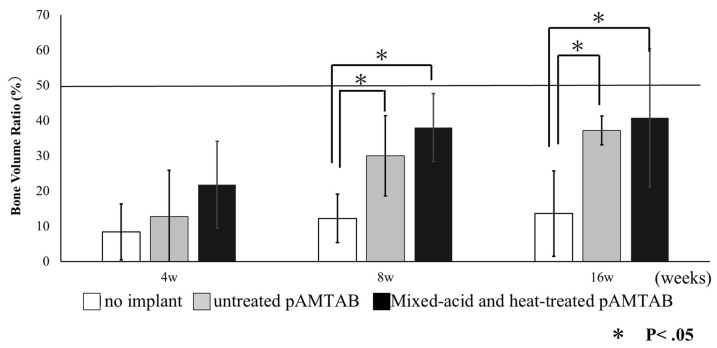
Bone volume ratio in all experimental groups. At 4 weeks, no significant difference is found among the three groups, but at 8 and 16 weeks, bone formation is significantly greater in the untreated porous additive-manufacturing titanium artificial bone (pAMTAB) group and mixed-acid and heat-treated pAMTAB group than in the no implant control group. The data for each group were analyzed by one-way analysis of variance and Tukey’s multiple comparison test using JMP^®^ software (SAS Institute Inc., Cary, NC, USA). *p* values < 0.05 were considered significant.

**Figure 8:**
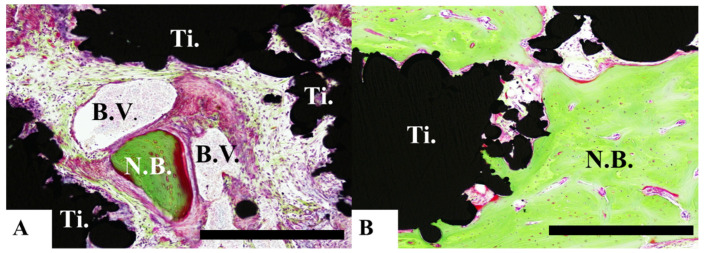
Histological analysis of mixed-acid and heat-treated porous additive-manufacturing titanium artificial bones (pAMTABs) at 16 weeks. (**A**): Blood vessels around newly formed bone and pAMTAB. (**B**): Active bone formation is observed partially. Scale bar indicates 100 µm. Ti: pAMTAB; Bo: calvarial bone; B.V.: blood vessels.

Bone formation around the pAMTABs was histologically evaluated. Although no significant differences between the groups were observed at 4 weeks after surgery, analyses at 8 and 16 weeks after surgery showed significantly greater bone formation in the untreated pAMTAB and mixed-acid and heat-treated pAMTAB groups than in the no implant control group. In addition, although bone occupancy tended to be greater in the mixed-acid and heat-treated pAMTAB group than in the untreated pAMTAB group, the difference was not significant. Furthermore, new blood vessel formation was observed within the pAMTABs (Figure 7 and Figure 8).

In the no implant group, no rat exhibited a bone formation score of 2.3 at any time point. No individual rats in the control group exhibited bone formation that exceeded a score of 1, and bone formation exceeding a score of 2 was observed in both the untreated pAMTAB and mixed-acid and heat-treated pAMTAB groups (Table 1).

## 4. Discussion

Bone-like tissue scaffolds from various structures have been investigated [9]. The present study aimed to develop a pAMTAB with an outer structure similar to that of the cortical bone and an inner structure similar to that of the cancellous bone. The cancellous bone has a honeycomb structure, each of which intersects at a right angle. Kock [10] reported that the results of stress analyses at the center of the femur, as well as the sequence pattern in the internal bone architecture, were consistent with stress distribution. In addition, the porous structure of additively manufactured Ti implants prepared using SLM technology produces an anisotropic strength similar to that of the human cancellous bone. Consequently, Matsushita et al. [5] reported that SLM technology enables the reproduction of a structure similar to that of the cancellous bone. To develop a pAMTAB with an outer structure similar to that of the cortical bone and an inner structure similar to that of the cancellous bone, additive-manufacturing technology, which enables the generation of bone with complex and arbitrary shapes based on STL data, was used. In the additive-manufacturing approach, a 30 μm-thick layer of Ti powder is selectively irradiated with a laser beam, leading to melting and subsequent hardening of the irradiated powder. Repetition of this process on multiple layers allows for the formation of a highly precise and accurate pAMTAB. Otawa et al. [11] previously reported a mean dimensional error of only 139 μm between their three-dimensional STL data and additively manufactured materials; an error of this magnitude posed no problem for use of the materials in jaw bone reconstruction. Porous Ti artificial bones with features similar to those of natural bones are considered potential material for use in jaw bone reconstruction [12]. Previous reports have also shown that porous Ti artificial bones are ideal bone replacement materials. The porous structure of Ti is believed to induce bone growth toward the inside, and as a result of fusion with the surrounding bones, porous Ti artificial bones are stable, which prevents the inserted implant from dropping out [12,13,14]. In the present study, newly formed bone and blood vessels were observed inside the porous material, indicating that the porous structure allows for the migration of cells inside the material. Furthermore, a previous study examining the implantation of flat-type additive-manufacturing Ti specimens into calvarial bones of rats reported no findings indicative of inflammation and good biological stability of the specimen [15].

Pattanayak et al. [6] reported that Ti has a pressure-resisting strength of 119.0 MPa at a porosity of 54.3% and 70.0 MPa at a porosity of 63.0%, indicating that its strength decreases with increasing porosity. As such, increasing the porosity of Ti could enable the formation of new bone inside the implant. In the present study, the porosity was set to 46% because, under such conditions, strength would be secured and formation of new bone inside the implant would be expected. In the future, we plan to conduct similar experiments to evaluate the effects of varying porosity and column diameter on the formation of new bone inside implants.

In the present study, the size of the bone defect in the rat skull was set to 5 mm. pAMTAB was supported by a fitting force for rat calvarial bone defects. Generally, an approximately 8 mm defect in rat calvarial bone is considered a critical defect [16,17]. However, to assess new bone formation inside the implant, a bone defect measuring < 8 mm was created in the present study to facilitate analyses. Furthermore, if a pAMTAB with a radius of 8 mm was to be inserted, fixation to the surrounding bones by the mechanical fitting force could be unreliable because of the curvature of the rat calvarial bone. Therefore, the size of the bone defect was set to 5 mm. No detachment of the pAMTAB was observed in any of the animals. In terms of the amount of bone formed and bone occupancy, the present findings indicated that the amount of bone formation that extended beyond the center of the bone defect was higher in the untreated pAMTAB group and mixed-acid and heat-treated pAMTAB group than in the no implant control group. It was demonstrated that pAMTAB promotes the formation of new bone both outside and inside the artificial bone in the membranous bone, such as the calvarial bone. This suggests that custom-made Ti devices with a porous structure are suitable materials for promoting new bone formation.

The effect of chemical treatment on the pAMTAB surface was also evaluated. In a previous study, Kokubo [7] evaluated various heat-treated temperatures after acid treatment. A surface roughness was produced on the Ti metal sample by the acid treatment, and this remained unchanged up to 600 °C, but the sample surface roughness decreased above 700 °C. Yamaguchi dipped mixed-acid and heat-treated Ti into simulated body fluid and then assessed its effect on bone formation [18]. In addition, Yamamoto et al. [19,20] created a small bone defect in rat calvarial bones and implanted Ti mesh that had been subjected to various surface treatments into the defect. They found significantly greater bone formation with Ti specimens that had been subjected to mixed acid and heat treatment. However, no studies have evaluated bone formation after implantation of mixed-acid and heat-treated pAMTABs in bone defects in vivo. In this study, bone formation tended to be greater with implantation of mixed-acid and heat-treated pAMTABs than with untreated pAMTABs, but the difference was not significant. The present findings indicate that the increased effectiveness of surface-treated Ti could be related to an increase in the area available for contact with bone. In future studies, we will examine the effects of varying the pore diameter, porosity, and size of the bone defect. In the present study, pAMTABs implanted in rat calvarial bone defects exhibited high bone formation. It is hypothesized that the surface texture of the pAMTAB contributed to the active formation of bone in the defect, and that mixed acid and heat treatment also promoted bone formation. However, additional studies are required to confirm that artificial bone custom made to fit bone defects integrates with the bone. Such studies could further contribute to the development of artificial bones suitable for use in reconstructive surgery.

## 5. Conclusions

By integrating mixed acid and heat treatment with additive-manufacturing technology based on the SLM method, pAMTABs were prepared, and their usefulness was evaluated by implanting the devices in rat calvarial bone defects and monitoring the formation of new bone. The present findings suggest that pAMTABs can be used as artificial bone which is similar to bone marrow-like structures in jaw bone reconstruction. It was considered to be one of the effective reconstruction methods where continuity of the jawbone has been lost due to a tumor. The technology used in the present study could facilitate further advancements in jaw bone reconstruction techniques.

## Figures and Tables

**Table 1:** Evaluation of bone formation in each group: In the no implant control group, no individual rats showed any bone formation exceeding a score of 1 at any time point, but in the porous additive-manufacturing titanium artificial bone (pAMTAB) groups, bone formation exceeding a score of 2 was observed in both the untreated pAMTAB and mixed-acid and heat-treated pAMTAB groups.

		Score				
	Weeks		0	1	2	3
no implant	4w (n = 5 each)		++++	+		
untreated pAMTAB			+++	++		
Mixed-acid and heat-treated pAMTAB			+	++	++	
no implant	8w (n = 5 each)		++	+++		
untreated pAMTAB				++	+++	
Mixed-acid and heat-treated pAMTAB				++	+++	
no implant	16w (n = 5 each)		+	++++		
utreated pAMTAB				+++	++	
Mixed-acid and heat-treated pAMTAB					++++	+

## Data Availability

The data presented in this paper is available with request from the corresponding author.
